# The aortic root, do we see the ‘hole’ picture?

**DOI:** 10.1007/s12055-024-01755-0

**Published:** 2024-06-01

**Authors:** Georgios Belitsis, Jonathan Robert Finch

**Affiliations:** 1 Department of Children’s Cardiovascular Disease, Institute of Cardiovascular Science, University College London, London, UK; 2 Department of Cardiothoracic Surgery, Great Ormond Street Hospital for Children, London, UK; 3 Department of Cardiothoracic Surgery, Royal Brompton and Harefield Hospitals, Hill End Road, Harefield, London, UB9 6JH UK

**Keywords:** Aortic root, Heart development, Coronary development, Left ostial process, Valve sparing

## Abstract

The aortic root is the segment of the aorta between the left ventricular outflow tract and the sinotubular junction of the ascending aorta, and, on one level, is merely a tube, with a valve at its base, dynamic structures below it, and notable for having the life-limiting coronary arteries originate within its sinuses. However, we propose that the perception of the aortic root has been historically grossly over-simplified by virtue of a bias towards its internal aspect, in terms of coronary ostia and subvalvar relationships through the fibrous skeleton and in so-doing a myocardial component on the external aspect has all but been ignored. This myocardial mass, a component of the left ventricular free wall, is sometimes termed the ‘left ostial process’ but appears to be rarely, if ever, considered by anatomists, cardiologists, and surgeons alike. By virtue of its direct continuity to the aortic root and proximal left coronary artery, it may have unique roles and, at the very least, deserves greater recognition and investigation. Herein, we propose that it could play a crucial role in cardiac embryology including coronary dominance, and may afford a physiological advantage, to the extent that it may have been selected for in evolutionary terms.

## Introduction

Conventional teaching of the aortic root concentrates on the continuity with the left ventricular outflow tract (LVOT) and particularly the structures most at risk at aortic valve and root intervention, i.e. anterior mitral valve leaflet, membranous septum, and His bundle, and also the continuity of the fibrous skeleton. However, herein we make a case for a deeper understanding of the human aortic root, in terms of evolution as compared to lesser animals, a different perception of the alignment of the root relative to the boundaries of the LVOT, and, specifically a need for greater understanding of the contribution made to the physiology of the aortic root and left main coronary artery by the adjacent myocardial mass of the left ventricular (LV) free wall, the rarely cited ‘left ventricular ostial process’ (LVOP).

## ‘Conventional’ views of the aortic root

### Anatomical composition and relationships

**Fig. 1:**
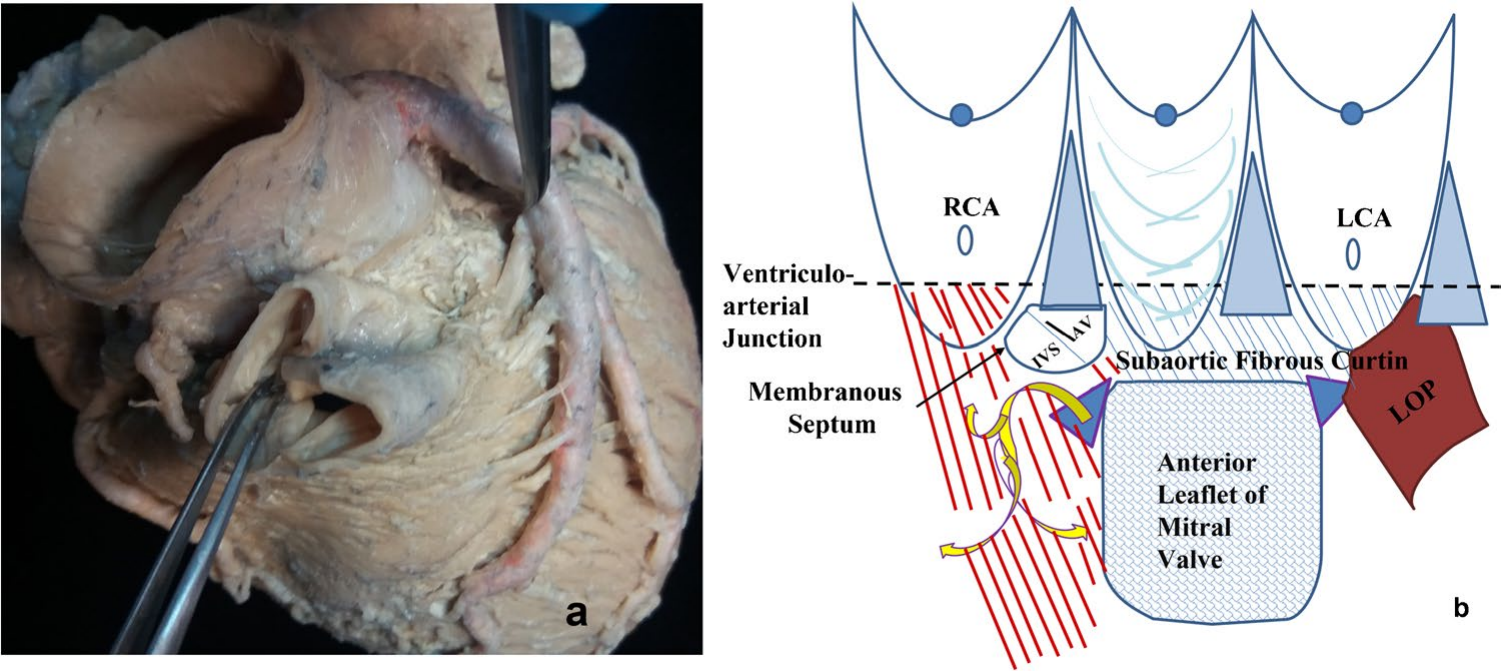
The ‘hole’ picture: **a** external aspect of intact aortic root, showing continuity with left ventricular outflow tract (LVOT) below and ascending aorta above, and **b** sketch of the internal aspect of incised aortic root (upon which current perceptions are heavily based). VA junction, ventriculoarterial junction; RCA, right coronary artery; LCA, left coronary artery; LOP, left ostial process. Illustration by Georgios Belitsis and accompanying photographs of human hearts from the cardiac archive, University College London

**Fig. 2:**
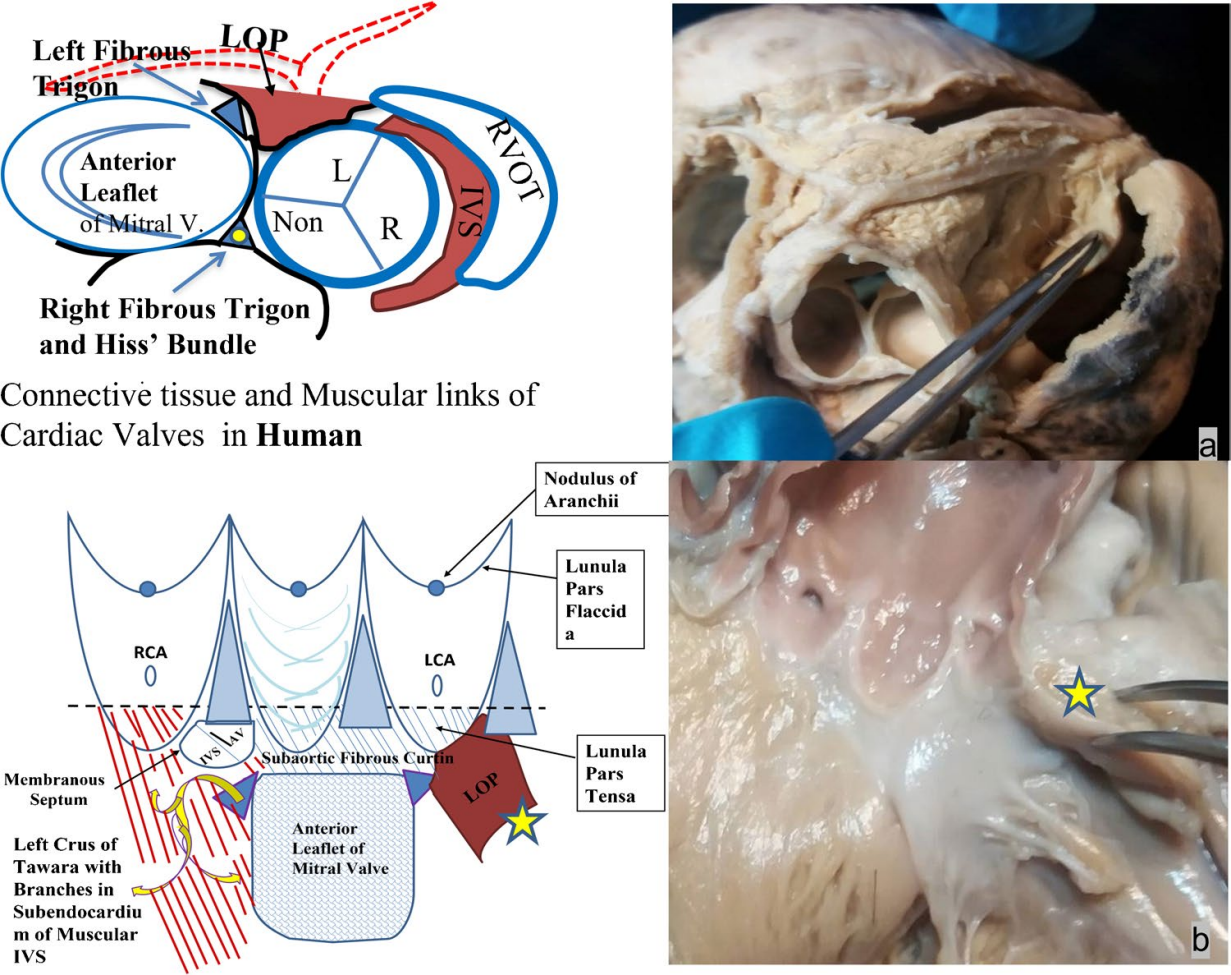
The ‘whole’ picture: **a** illustration and photo of the external aspect of intact aortic root, from above showing continuity with left ventricular outflow tract (LVOT) below and ascending aorta above, and **b** illustration and photo of the internal aspect of incised aortic root including the left ostial process (LOP). RVOT, right ventricular outflow tract; IVS, interventricular septum; RCA, right coronary artery; LCA, left coronary artery. Illustration by Georgios Belitsis and accompanying photographs of human hearts from the cardiac archive, University College London

The aortic root can be defined as that segment of the aorta that lies between the LVOT and the sinotubular junction of the ascending aorta. The proximal portion can be considered to ‘sit’ atop the highly complex LVOT, which is itself a composite of the interventricular septum (IVS), the aorto-mitral ‘continuity’ (AMC), and the portion of the LV free wall that supports the left aortic sinus, sometimes referred to as the ‘left ventricular ostial process’ [1] (LVOP) or simply ‘left ostial process’ (LOP) (Figs. 1 and 2).

### Physiological role

In conjunction with the aortic annulus, the aortic root is a highly dynamic structure which undergoes cyclic changes during the cardiac cycle [2].

### Spatial orientation and embryological origin

Pre-eminent anatomists and embryologists have adopted slightly differing foci regarding the developmental subtleties of the aortic root in normal and abnormal morphologies. R. Anderson focuses on the continuity of the ventriculoarterial junction (VAJ) (in contrast to a simplistic model of an ‘annulus’ represented by the nadir of the sinuses) instead describing a ‘crown’-like attachment of the aortic leaflets traversing both sides of the histological VAJ [2] and their relationship with the interleaflet triangles [3]. However, a case can be made, on developmental grounds, for regarding the AMC as an aorto-mitral *discontinuity* by virtue of the tissue plane between VAJ and the base of the mitral valve. Perforation of this plane, as can occur during resection of subaortic membrane, can take one ‘outside’ the heart, or increase the chance of LVOT aneurysm [4]. In developmental terms, the ‘scaffold’ of three-dimensional LVOT is the former sub-aortic conus, the embryological transition between the primordial ventricle and truncus arteriosus [5–7]. Richard and Stella Van Praagh wrote extensively on the fundamental differences in the sub-aortic conus in transposition of the great arteries (TGA) and congenitally corrected transposition of the great arteries (ccTGA) pathologies [7–10].

Just before the ‘D looping’ of the heart takes place, the atrioventricular (AV) junction connects only to the LV, while only the right ventricle (RV) leads to the outflow tracts. This initial arrangement places the LV far away from the outflow tract of the heart tube. During ‘D looping’, the LV effectively slides to the left and posterior to the AV junction, so that the RV occupies the anterior part of the AV connection. This can be considered a ‘fight’ for finite space, with the RV ‘granted’ this access to the AV junction. Failure to do so results in a double inlet LV pathology. Simultaneously, the LV gains access to the posterior part of the truncus arteriosus, thus forming an outlet for itself, such that failure to do so results in a double outlet RV morphology. To allow this, the sub-aortic conus needs to shrink (or at least not grow further) in order that the LV part of the AV junction will appear immediately under the posterior placed LV outflow tract. This way the RV will be allowed the anterior portion of the AV junction and the LV will be allowed the posterior portion of the truncus arteriosus. Hence, just days later, the aortic root will find itself ‘wedged-in’ between mitral and tricuspid valves, being pulled downwards by the fusion of the truncal cushions. In contrast, with abnormalities of this process, such as in endocardial cushion defect, the aortic root remains in a higher position, effectively sitting on top of an elongated LVOT. This same ‘traction’ effect, invaginating the root into the LVOT, results in the formation of the tripartite fibrous valvular skeleton from hitherto unrelated components.

## A ‘new’ view of the aortic root that recognises the left ostial process and its potential roles

### Anatomical composition and relationships

The LOP is the cranial-most aspect of the LV free wall, in direct continuity with the left coronary sinus and forming the floor of the proximal left coronary artery (Fig. 2a).

Further note that the right-hand edge of the opened root is merely inverted beneath the left coronary sinus so as to illustrate the normally hidden LOP on its external aspect (Fig. 2b).

**Fig. 3:**
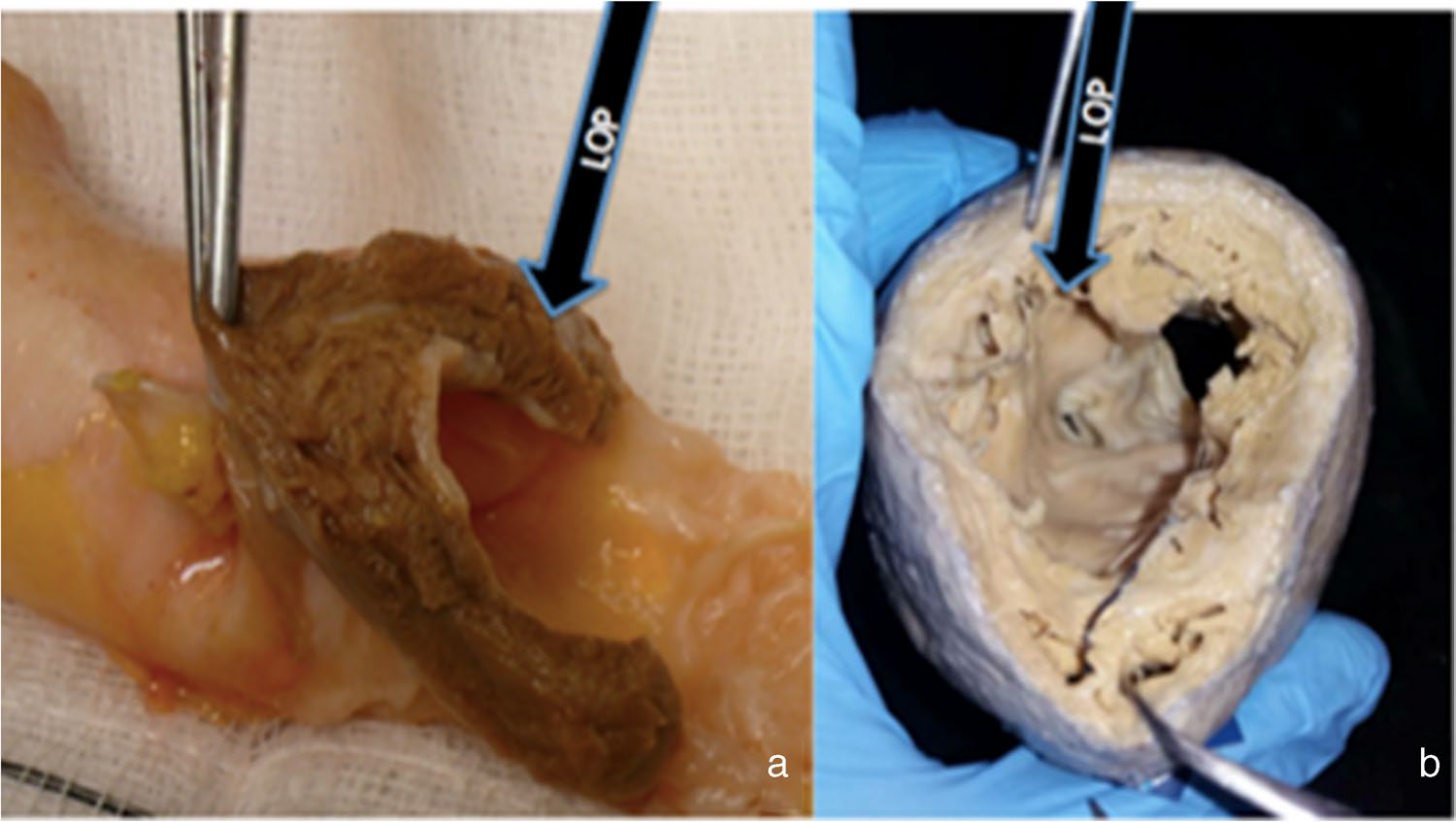
**a** Homograft aortic root and **b** human heart transacted at mid papillary level with left ventricular outflow tract (LVOT) and left ventricular process (LOP) seen*.* **a** Courtesy of Mr Jonathan Finch and **b** photograph of human heart from the cardiac archive, University College London

The myocardial mass of the LOP is seen from the unparalleled view afforded by an explanted aortic root, in the form of an aortic root homograft (Fig. 3), and from an anatomical human heart specimen where the LOP is seen from the apex. We hypothesise that this normally hidden component is wrongly overlooked in not only anatomical terms but also surgical, contractile-physiological, synthetic-paracrine, and embryological terms.

### Potential role of the LOP in aortic root physiology

One could hypothesise that the LOP could afford biosynthetic machinery for autoregulation of the aortic root annulus, affecting mechanics and regeneration.

Firstly, there is clear evidence for a downward motion of the aortic root in systole and also a straightening of the ventriculo-aortic angle, thus increasing the LVOT orifice area. The aortic media will doubtless afford an elasticity element to this cyclical occurrence but a greater focus on the LOP might add an additional active, contractile component to the argument [11–13].

It may contribute to a more oblique offset of the aortic root from the LV, and changing both the aortico-mitral angle and the way the aortic root is positioned posteriorly to the right ventricular outflow tract (RVOT) maximising the asymmetry of flow dynamics in the heart [14].

As a second example, take the surprising finding of annular contraction in some patients after the Yacoub remodelling aortic valve-sparing root replacement operation, as compared to the later David re-implantation aortic valve-sparing root replacement operation. The fundamental difference between the two is the lack of an external, circumferential restrictive Dacron tube around the aortic annulus in the former. It is often suggested that by having the external restrictive Dacron tube, only the David re-implantation operation can achieve annular stability whereas conversely, the same simplistic view would contend that, without the bioprosthetic support, the Yacoub remodelling operation will invariably fail by virtue of lack of annular stability, allowing the annulus to dilate and the native aortic valve to become insufficient. Such a view either disregards or downplays the potential for autoregulation of root geometry.

Note that while the prepared aortic root with part-resected sinuses is comparable in the two, the Yacoub procedure has only an interdigitating/incomplete Dacron tube, in contrast to the David re-implantation procedure which has a complete circumferential ring of Dacron tube surrounding the aortic annulus.

## An evolutionary argument for the physiological role of the LOP, whereby the LOP interrupts the complete fibrous continuity of the valvular skeleton of the heart

One can consider that the formation of the tripartite fibrous skeleton excludes epicardial continuity (*and by extension, electrical continuity*) with ¾ of the aortic root circumference, leaving only the epicardium of the LOP in contact with it.

**Fig. 4:**
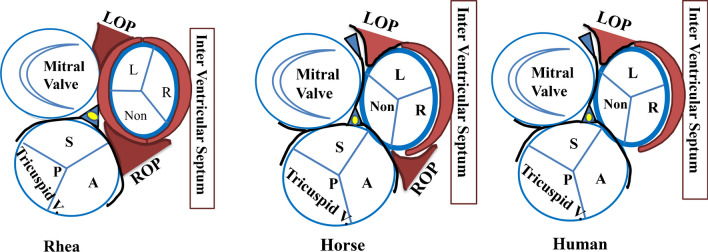
Comparative soft tissue interruption of the valvular skeleton of the Rhea, horse, and human. Note the variability of the continuity of the fibrous skeleton of the heart in Rhea, horse, and human. LOP, left ostial process; ROP, right ostial process; IVS, interventricular septum. Illustration by Georgios Belitsis

In contrast to humans, analysis of many other species reveals fundamental differences in the degree of fusion of the three valves and the nature of the connective tissue between them as a result (Fig. 4) (reference from McAlpine book) [15]. At the other end of the spectrum is the Rhea, whose valves remain near separate entities, separated by the myocardium, and, therefore, both left and right ostial processes, joined by a muscular bridge. The equine root also sits atop the right and left ostial processes but its aortic and mitral valves are closer together, and consequently the muscular bridge has been replaced by a fibrous AMC. We hypothesise that this variability of valvular relationship and secondary fibromuscular differentiation of the bridging connective tissue might be an evolutionary process. In human evolution, it can be argued that the right ostial process has been replaced by the right fibrous triangle, but with the LOP remaining and effectively ‘bridging’ the gap between left fibrous triangle (left hinge margin of the mitral valve) and muscular IVS.

Taking such an ‘evolutionary process’ hypothesis a step further, one could argue that evolution could erase the LOP altogether, ‘allowing’ the entire perimeter of the LV base (‘*ostium*’) to be shared between mitral and aortic valves, thus maximising inlet and outlet surface areas.

But, conversely, one can also make an argument for evolution having preserved the LOP in humans for physiological gain. Conventional teaching of aortic root relationships almost completely overlooks the LOP in comparison to AMC and IVS but it should not be forgotten that the LOP is the *only* direct connection of morphological primordial LV musculature to the aortic wall. In contrast, at a cellular level, the remainder of the aortic root connections are derivatives of the truncal ridges (neural crest cells) fusing with the IVS crest and fibrous AMC (representing the reabsorbed conus).

### Potential role of the LOP in coronary artery development and physiology

Quite apart from a potential relationship between the LOP and aortic root physiology one can readily make an argument for a relationship between it and the physiology of the left coronary artery, which it supports and secondarily the development of the left coronary artery, and indirectly the right coronary system.

### The LOP and the proximal left coronary artery, an overlooked spatial relationship?

**Fig. 5:**
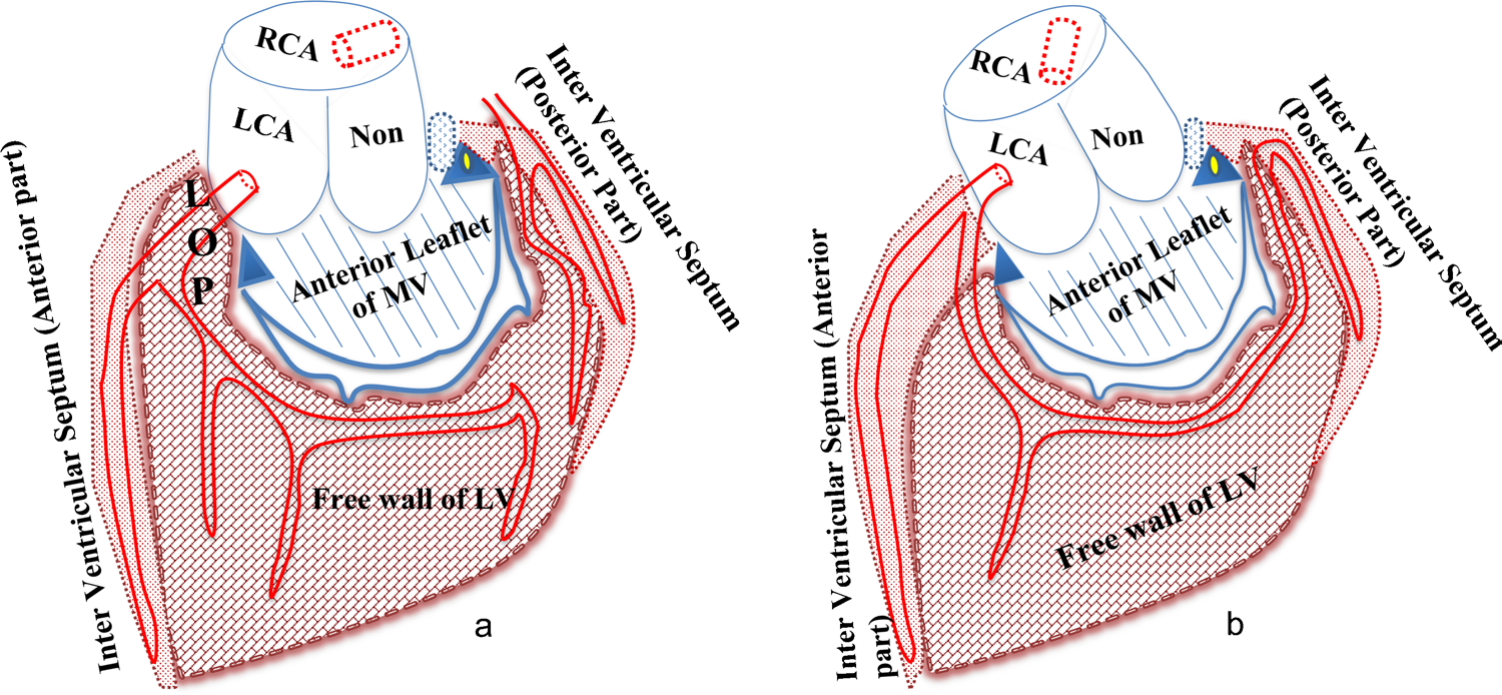
The integral relationship between the left ostial process (LOP) and length of the left main stem (LMS). Illustration by Georgios Belitsis

The length of the left main stem (LMS) is, by definition, the distance from the ostium in the left aortic sinus to the left side of the AV groove and anterior interventricular (IV) groove (Fig. 5). The LOP is what separates the anterior IV groove from the posterior AV groove and its size and shape are integral to this epicardial geometry *since, as previously described, the LOP effectively lifts and angles the root away from the AV groove and anterior IV groove*. Correspondingly, it follows that if the left aortic sinus sit on top of a large LOP, the LMS will be long. Conversely, if the aortic root is not seated on the LOP, and hence the left fibrous trigone effectively reaches the IVS, the LMS will be short.

### The LOP and the proximal left coronary artery, an overlooked physiological relationship?

The anatomical relationship between LOP, the LMS, and ramus intermedius (where present) is beyond doubt. Far more than merely forming the floor of the LMS, connecting the aortic root to the left anterior descending and circumflex vessels, we hypothesise that the greater the mass of LOP in continuity with the LMS, the greater the degree of extrinsic control of vasoactivity and mechanical protection, thus affording an evolutionary advantage*?*

We propose that left dominance, with shorter LMS, and hence lesser continuity with the LOP, was negatively selected against. With right dominance strongly prevailing in the current era, we hypothesise that this 70% have the advantage of twin LV blood supply and a LMS that is more heavily regulated by paracrine molecular signalling from the large LOP muscle mass forming the floor of its course*.*

### Evidence from computer tomography (CT) coronary angiography

We retrospectively reviewed coronary angiograms and CT coronary angiograms in 146 adult patients being investigated prior to transcatheter aortic valve implantation (TAVI), in which measurement of coronary ostial height is a key observation at a planning stage. Of these 146 patients, 120 were right dominant, 22 left dominant, and 4 were co-dominant.

This correlation between shorter LMS length and left dominance was described but our own observations suggest also a lower incidence of a ramus intermedius vessel in this minority. After directly measuring the LMS, we noticed that shorter LMS (12 mm for left coronary artery (LCA) dominance while 13.84 mm was the average LMS length for all hearts and 14.06 mm for right coronary artery (RCA) dominant hearts) was related to LCA dominance [16]. The length of the LMS was increasing (average of 14.48 mm) when it was trifurcating. Trifurcation occurred in 47% of all hearts but only on 36% of LCA dominant hearts. The number of obtuse marginal (OM) branches was also reducing with LCA dominance.

## A ‘finite embryological cellular pool’ hypothesis would further support the observation of a long LMS and a non-LCA dominant coronary system

It has been suggested that there is a finite pool of embryological vascular endothelial cells, and one which nature seeks to recycle for new roles after programmed vascular involution. Take, for example the apparent recycling of the defunct right umbilical vein at only 3 weeks gestation, with cells seemingly recycled into liver sinusoids.

Similarly, if one considers that the pool of endothelial cells available for coronary vasculogenesis is similarly limited, then the presence of a longer LMS and, by extension, ramus intermedius, places limitations on those cells available to form the coronary arterial network towards the posterior IV sulcus, and right dominance results. Taking this a step further, it could be hypothesised that this vasculogenesis of a long LMS could fall under the control of molecular and cellular signalling derived from the endocardium and myocardium of the LOP.

The LOP does play a dual role on the development of the LCA. It influences the flow within the LMS by strong and early paracrine stimuli, offering possibly the best way of regulation of flow in developing of the early left ventricle. Additionally, its spatial presence places the LCA ostium away from the IV and AV grooves necessitating a long LMS to fill the gap, with early consumption of the finite number of endothelial cells. Thus, the LOP remains preciously maintained by the evolutionary process as it facilitates the twin-dual coronary artery blood supply of the left ventricle through RCA dominance.

## The evolutionary steps to a circulation in series and the need of coronary artery supply for the newly added subpulmonic ventricle

The oxygen and nutritional supply of the myocardium is not based on coronary arteries that connect to the arterial outflow of the heart, in all species [17]. Transmyocardial supply exists in many fish, having a non-compacted myocardium. The first coronary arteries developed possibly 500 million years ago to fish while parts of the outer myocardium were becoming more compact [18]. Transmyocardial oxygenation from the common heart chamber, combined with branchial (fish) or transdermal (amphibians) gas exchange, was abundant, as fully terrestrial species evolved. Avian species, reptiles, and mammals [19], exchanging gasses on the lungs, require a subpulmonic ventricle, with a progressively more compact myocardium that necessitated coronary circulation. Evolving further, the blood in the heart was not getting mixed any more (either by functional septation due to two distinct columns of laminar flow) in lower vertebrates or by complete anatomic septation in higher mammals. Consequently, the role of a transmyocardial blood supply would be limited in supplying the pre-pulmonic ventricle when the pulmonary circulation and systemic circulation are in series (i.e. the blood in the pre-pulmonic ventricle would have very low saturations, not sufficient to supply is own wall). The dual coronary artery supply has been present as early as in fish [20]; it would require lots of evolutionary modulations to meet the demands of higher species and especially mammals, while exercise and homeostasis including the homoeothermia. Having an additional pumping chamber evolving further and positioned rostral to the systemic ventricle, the anterior coronary artery has been offered for the supply of the subpulmonary ventricle, at least for the majority of its course, maintaining its commitment in supplying the posterior part of the IV in most of us. It takes a glance at a heart for one to tell that the two ventricles are really not looking alike. How different do the coronary arteries that supply each ventricle really are?

### RCA observations

The LCA ‘crawling’ on the myocardium is giving the septal perforators very early on its course. The myocardium itself by its squashing effect along systole is influencing the flow in the LCA on its very early segments, necessitating the maintenance of adequate lateral pressures within the LCA sinus of Valsalva, to prevent reversal of flow within the vessel. This possibly explains the need of a rather constant position of the LCA orifice within the sinus, observed in our study. Undoubtedly, the RCA flow is also influenced to some extent, by the squashing effect of the myocardium and obviously by the arteriolar vaso-reactivity. The RCA along its course in the AV groove is maintaining an anatomic and possibly physiologic distance from the myocardium. All the major proximal branches are running epicardially and the perforators of them are penetrating thin and well-trabeculated RV muscle. The RCA will get on close contact with the myocardium only when (and if) it reaches the post IV groove, following the archaic arterial-myocardial relation of the LCA system. *We hypothesised that the spatial relation of the RCA and LCA to their corresponding myocardium could be tracked back to their origin from the aortic root.*

In the above-mentioned pool of human hearts, we additionally observed a trend towards higher positioning of the right coronary ostium with right coronary dominance: 13.6 mm above the annulus for left dominant hearts and 14.48 mm for all hearts and 14.50 mm for right dominant hearts, but with the difference not reaching statistical significance (perhaps owing to the small number of the former group). The height of the LCA ostium did not show a great amount of variation in our study population.

Despite the statistical insignificance, we could see that the RCA becomes dominant when it finds its origin, away from the IVS, and positioned high up with the sinus of Valsalva. Possibly, this implies that the artery to the anterior ventricle is favoured by direct exposure to aortic wall hemodynamics (being an artery perfused on systole) and staying away from the IVS mechanics and the systemic ventricle vasoreactivity.

### Limitations

To be able to identify the role of the LOP on the governance of local aortic root embryogenesis, further observations will be needed. We have attempted to declare that the LOP, being a large and the only part of the LV connecting to the aortic wall, should play a role in the embryogenesis of the aortic root. We used CT data available to demonstrate relationships in three-dimensional (3D) space. The limited number of CT scans available and the resolution that conventional clinically used CT is offering suggest that there is a need of further studies. Soon, relevant data may well derive from different scientific modalities. The rapidly developing high-resolution micro-CT of the heart and synchrotron-based X-ray phase contrast imaging will allow a much more detailed look in the aortic root architecture. Data sets deriving from different stages of embryogenesis of animal models can be analysed with the above modalities and more light can be brought in the field.

## Discussion

We suggest that the evolutionary modulation of the human subaortic conus possibly has affected the finite development of the proximal coronary artery themselves, influencing adequate and simultaneous supply to both pumping chambers. The complete absorption of the right ostial process (ROP), wedges the aorta in-between, the tricuspid and mitral valves exactly at the level of the anterior AV groove, where the main branches of the vascular networks that supply the subpulmonary myocardial field, would be. Having the aortic root being pulled down by the absorption of the ROP allows the right ventricular main branch to be implanted easily to the arterial outflow sinus, at embryonic stage 18 [21]. The presence of a LOP on almost opposite side of the VAJ would be acting as hypomochlion, wedging even further the aortic root, and possibly tilting it, allowing the RCA ostium to be implanted even higher in the RCA sinus to be. This way, the distal RCA at its very terminal branch in the posterior IV groove or posterior LV wall would be under systemic ventricle influence while its proximal course flow would be facilitating the perfusion of the subpulmonary ventricle that is somehow a younger addition on the evolutionary course of the cardiorespiratory bio-machinery.

## Data Availability

The data analysed and available to the journal upon request is completely anonymised.

## Declarations

### Human tissue authority

The hearts that appear on the photos are from the cardiac archive of University College London (UCL). Institute of Child Health’s Human Tissue Authority research committee also falls under the wider governance of the UCL Human Tissue Authority research committee.

## References

[1] McAlpine WA. Heart and coronary arteries. An anatomical atlas for clinical diagnosis, radiological investigation, and surgical treatment. Berlin: Springer-Verlag, 1995. 1975.

[2] Anderson RH. Clinical anatomy of the aortic root. Heart. 2000;84:670–3. 11083753 10.1136/heart.84.6.670 PMC1729505

[3] Sutton JP, Ho SY, Anderson RH. The forgotten interleaflet triangles: a review of the surgical anatomy of the aortic valve. Ann Thorac Surg. 1995;59:419–27. 7847960 10.1016/0003-4975(94)00893-C

[4] Yacoub M, Onuzo O, Riedel B, Radley-Smith R, Hanley FL. Mobilization of the left and right fibrous trigones for relief of severe left ventricular outflow obstruction. J Thorac Cardiovasc Surg. 1999;117:126–33. 9869766 10.1016/S0022-5223(99)70477-0

[5] Goor DA., Dische R, Lillehei CW. The conotruncus: I. Its normal inversion and conus absorption. Circulation [Internet]. 1972;46:375–84. Available from: http://circ.ahajournals.org/cgi/doi/10.1161/01.CIR.46.2.375. 10.1161/01.cir.46.2.375 5046031

[6] Anderson RH, Wilkinson JL, Arnold R, Lubkiewicz K. Morphogenesis of bulboventricular malformations. I. Consideration of embryogenesis in the normal heart. Br Heart J. 1974;36:242–55. 4824533 10.1136/hrt.36.3.242 PMC458826

[7] Goor DA, Edwards JE. The spectrum of transposition of the great arteries: with specific reference to development anatomy of the conus. Circulation. 1973;48:406–15. 4726219 10.1161/01.CIR.48.2.406

[8] Pasquini L, Sanders SP, Parness IA, Colan SD, Van Praagh S, Mayer JE, et al. Conal anatomy in 119 patients with d-loop transposition of the great arteries and ventricular septal defect: an echocardiographic and pathologic study. J Am Coll Cardiol. 1993;21:1712–21. 8496542 10.1016/0735-1097(93)90392-E

[9] Anderson RH, Spicer DE, Mohun TJ, Hikspoors JPJM, Lamers WH. Remodeling of the embryonic interventricular communication in regard to the description and classification of ventricular septal defects. Anat Rec. 2019;302:19–31. 10.1002/ar.24020 30408340

[10] Van Praagh R, Pérez-Treviño C, López-Cuellar M, Baker FW, Zuberbuhler JR, Quero M, et al. Transposition of the great arteries with posterior aorta, anterior pulmonary artery, subpulmonary conus and fibrous continuity between aortic and atrioventricular valves. Am J Cardiol. 1971;28:621–31. 5124722 10.1016/0002-9149(71)90049-X

[11] Thubrikar M, Bosher LP, Nolan SP. The mechanism of opening of the aortic valve. J Thorac Cardiovasc Surg. 1979;77:863–70. 439922 10.1016/S0022-5223(19)38191-7

[12] Leyh RG, Schmidtke C, Sievers HH, Yacoub MH. Opening and closing characteristics of the aortic valve after different types of valve-preserving surgery. Circulation. 1999;100:2153–60. 10571974 10.1161/01.CIR.100.21.2153

[13] Beller CJ, Labrosse MR, Thubrikar MJ, Szabo G, Robicsek F, Hagl S. Are there surgical implications to aortic root motion? J Heart Valve Dis. 2005;14:610–5. 16245499

[14] Kilner J, Yang Z, Wilkes A, Mohiaddin R, Firmin D, Yacoub M. Asymmetric redirection of flow through the heart. Nature [Internet]. 2000;404:759–61. Available from: 10.1038/35008075. 10.1038/35008075 10783888

[15] McAlpine WA. Heart and coronary arteries. 1st ed. Heidelberg: Springer-Verlag; 1975. pp. 27–28.

[16] Gauss S, Pflederer T, Marwan M, Daniel WG, Achenbach S. Analysis of left main coronary artery and branching geometry by coronary CT angiography. Int J Cardiol. 2011;146:469–70. 21144604 10.1016/j.ijcard.2010.10.140

[17] Reese DE, Mikawa T, Bader DM. Development of the coronary vessel system. Circ Res. 2002;91:761–8. 12411389 10.1161/01.RES.0000038961.53759.3C

[18] Ostadal B, Ostadalova I, Dhalla NS. Development of cardiac sensitivity to oxygen deficiency: comparative and ontogenetic aspects. Physiol Rev. 1999;79:635–59. Available from: http://www.ncbi.nlm.nih.gov/pubmed/10390514. Accessed 12 May 2024. 10.1152/physrev.1999.79.3.635 10390514

[19] Kul'chitskiĭ KI, Romenskiĭ OIu. Evoliutsiia krovenosnykh sosudov stenki serdtsa [Evolution of blood vessels of the heart wall]. Arkh Anat Gistol Embriol. 1986;90:8–16. Russian. 3954607

[20] Victor S, Nayak VM, Rajasingh R. Evolution of the ventricles. Tex Heart Inst J. 1999;26:168–75; discussion 175–6. Available from: http://www.pubmedcentral.nih.gov/articlerender.fcgi?artid=337097&tool=pmcentrez&rendertype=abstract. Accessed 12 May 2024. PMC337097 10524737

[21] Hutchins MG, Kessler-Hanna A, Moore GW. Pathophysiology and natural history development of the coronary arteries in the embryonic human heart. Circulation. 1988;77:1250–7. 3286038 10.1161/01.CIR.77.6.1250

